# Fungal Communities in the Native New Zealand Medicinal Plant *Pseudowintera colorata* (Horopito) Are Determined by Plant Organ Type and Host Maturity with Key Members Promoting Plant Growth

**DOI:** 10.3390/microorganisms9122576

**Published:** 2021-12-13

**Authors:** Neeraj Purushotham, Eirian Jones, Jana Monk, Hayley Ridgway

**Affiliations:** 1 Department of Pest-Management and Conservation, Faculty of Agriculture and Life Sciences, Lincoln University, Lincoln 7647, New Zealand; eirian.jones@lincoln.ac.nz (E.J.); Hayley.ridgway@plantandfood.co.nz (H.R.); 2 AsureQuality, Lincoln 7647, New Zealand; Jana.monk@outlook.com; 3 The New Zealand Institute for Plant and Food Research Limited, Christchurch 7608, New Zealand

**Keywords:** plant-microbe interactions, endophytes, endophytic fungi, plant pathogens, microbial ecology, DGGE, medicinal plant, biocontrol, antifungal activity, New Zealand

## Abstract

The plant *Pseudowintera colorata* is well known for its antimicrobial and medicinal properties and is endemic to New Zealand. Using PCR-Denaturing gradient gel electrophoresis (DGGE), we investigated the factors influencing the composition of endophytic fungal communities in *P. colorata* from ten distinct sites across New Zealand. Our results showed that plant organs of *P. colorata* influenced the diversity and richness of endophytic fungi (PERMANOVA, *p* < 0.05). In addition, plant maturity and its interactions revealed that endophytic fungal communities formed discrete clusters in leaves, stems, and roots of mature and immature *P. colorata* plants (PERMANOVA; *p* = 0.002, *p* = 0.001 and *p* = 0.039, respectively). For identifying isolates with biocontrol potential, dual culture tests were set up against four different phytopathogenic fungi. Isolates with high activity (zone of inhibition > 10 mm) were sequenced and identified as *Trichoderma harzianum*, *Pezicula neosporulosa*, *Fusarium* *tricinctum*, *Metarhizium* sp., and *Chaetomium* sp. Applying selected endophytic fungi (*n* = 7) as soil drenchers significantly increased the growth of *P. colorata* seedlings and produced more internodes. Seedling shoots treated with *Trichoderma* sp. PRY2BA21 were 2.2 × longer (8.36 cm) than the untreated controls (3.72 cm). Our results elucidate the main plant factors influencing fungal community composition and demonstrate a role for endophytic fungi in *P. colorata* growth and further demonstrate that medicinal plants are a rich source of endophytes with potential as biocontrol agents.

## 1. Introduction

Almost all land plants are inhabited by endophytic microorganisms, including fungi [1]. Endophytes are microbes that colonize inner plant tissues without causing any apparent symptoms of disease, and in return for nutrients and habitat, some can confer beneficial traits to the host plant, which include growth promotion, tolerance to biotic and abiotic stress, and biological control of phytopathogens [2,3,4]. Endophytic fungi have been identified for their potential to synthesize a wide variety of biologically active compounds, including the same or similar compounds for which the host plant is recognized [5,6,7]. For example, *Taxomyces andreanae*, an endophytic fungus, and its host, *Taxus brevifolia* (Pacific yew tree), are both reported to produce paclitaxel (Taxol^®^), an anticancer compound [8]. Similarly, the anticancer drug camptothecin, the anticancer drug lead compound podophyllotoxin, and the natural insecticide azadirachtin are co-produced by the plants and their associated endophytic fungi [9,10,11,12].

In addition to being rich sources of bioactive compounds, native medicinal plants also host unique endophytic fungi with potential applications in treating infectious diseases, as biocontrol agents, and for several other applications, thus harnessing the potential of endophytic fungi in medicinal plants is important [13]. *Pseudowintera colorata* (horopito) is a primitive endemic medicinal shrub that grows in the sub-alpine regions of New Zealand and has been an integral part of traditional Māori medicine (Rongoā). The leaves of *P. colorata* have been used as a remedy for fever, toothache, skin infections, and gonorrhea, and contain two sesquiterpene dialdehydes, polygodial and 9-deoxymuzigadial [14,15,16]. Polygodial has been reported to possess potent antifungal and antibacterial properties [17,18]. Previous studies on *P. colorata* have assessed the community structure and functional potential of endophytic bacteria and actinobacteria, but did not investigate endophytic fungi, which may confer unique characteristics to the host with a key influence on plant growth, development, and disease resistance [19,20]. For example, the endophytic fungus *Piriformospora indica* when colonizing the roots of *Prosopis juliflora* (mesquite) and *Zizyphus mummularia* conferred biotic (resistance against root pathogens) and abiotic resistance (salt stress) to its plant hosts [21,22]. Although there is increasing evidence highlighting the importance of endophytic fungi from medicinal plants, there is a significant knowledge gap regarding the endophytic fungi in native New Zealand medicinal plants.

While the use of medicinal plants as a source of biologically active compounds has been documented by ancient agricultural societies, the diversity of the endophytic fungi among the medicinal plants is poorly understood [23,24]. Endophytic fungi can directly or indirectly influence plant growth and productivity and, as with international research, the endophytic fungi of *P. colorata* may function in enhancing the growth of the host plant and protect against phytopathogens. For example, the colonization of maize plants by the endophytic fungus *P. indica* led to increased growth and systemic resistance to the root pathogen *Fusarium verticilloides* by enhancing antioxidant defenses within the host plant [25]. Other research has demonstrated that endophytic fungi promote the growth of plants by secreting plant growth regulators, enhancing hyphal growth and mycorrhizal colonization, by producing siderophores, and fixing nitrogen [26,27]. A study on the Indian medicinal plants *Withania somnifera* and *Spilanthes calva* demonstrated that inoculation with *P. indica* significantly increased the growth and yield [28]. Further research has elucidated that this molecular mechanism, via the activation of specific kinase proteins (MAPK3/6), is involved in plant growth [29]. The leaves of *P. colorata* are used for the extraction of polygodial to manufacture Kolorex^®^, a treatment for candidiasis. However, as *P. colorata* is a very slow growing plant, the endophytic fungal inoculants may offer a solution to promote the growth of *P. colorata*, which could have a positive impact on the ecology of the host and thus offer a sustainable solution to the industry as such. As the first study investigating the importance of endophytic fungi in *P. colorata*, this study has implications for future work on the ecology and biocontrol potential of endophytic fungi in native medicinal plants.

## 2. Materials and Methods

### 2.1. Sample Collection and Processing

**Figure 1:**
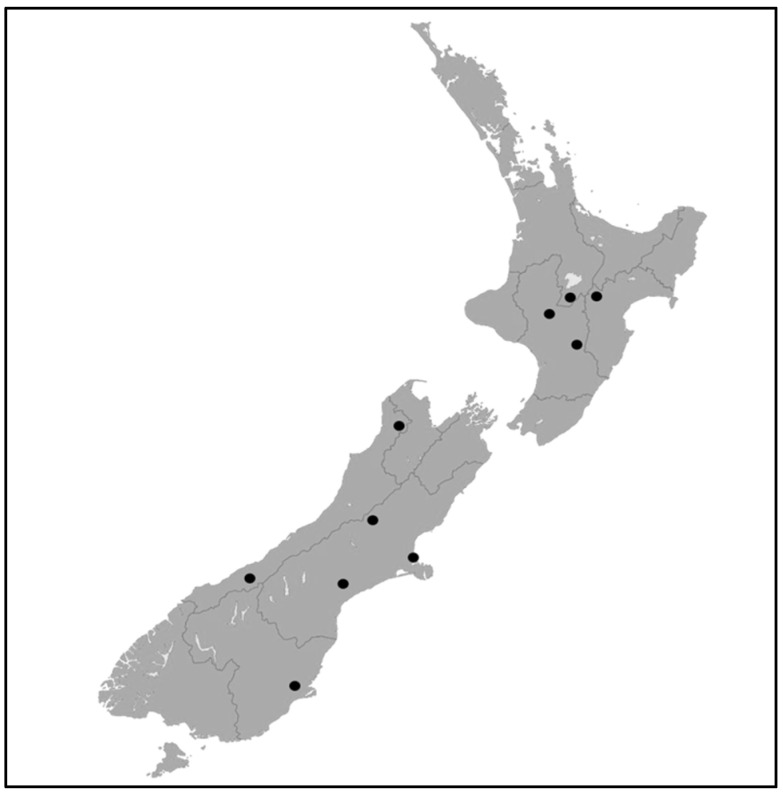
New Zealand map showing sampling locations (●).

*Pseudowintera colorata* plants were sampled between March and August 2014 from ten distinct sites across New Zealand, and a total of 87 individual plants (leaves, stems, and roots) were collected and processed as described previously (Table 1, Figure 1) [19]. Leaves sampled were fully open, mature, and free of herbivory or disease damage. Woody stems and lateral branches, including green succulent growth, were selected and cut using sterile secateurs. Roots were collected by excavating soil close to the *P. colorata* plant being sampled, and putative lateral roots along with root hair were traced back to the *P. colorata* plant and cut using sterile secateurs. To check the effectiveness of the sterilization process, leaves were imprinted onto synthetic nutrient deficient agar (SNA, SIFIN, Berlin, Germany) and R2A (Difco laboratories, Detroit, MI, USA) plates, and an aliquot of the final rinse water was plated onto SNA and R2A agar prior to sectioning the tissues. The plates were incubated at 25 °C for 24–48 h, and the absence of growth on plates indicated that surface sterilization was successful. The surface-sterilized tissues were then cut into 1-mm portions and plated onto SNA amended with ampicillin (100 µg/mL) to isolate fungi selectively. The plates were incubated at 20 °C for 5–7 d in 12 h light/12 h dark cycle, and the emerging mycelium was transferred to sterile potato dextrose agar (PDA, Difco laboratories, Detroit, MI, USA). Portions of each sterilized plant organ were stored in sterile glycerol at −80 °C to extract DNA for DGGE analysis.

### 2.2. Diversity Analysis of the Endophytic Fungi in P. colorata Using DGGE

Before extracting DNA, surface-sterilized *P. colorata* organs were treated with 1.25 µL of 20 mM propidium monoazide (PMA, Biotium, Fremont, CA, USA) to avoid amplification of epiphytic DNA by PCR [20,30]. DNA was extracted using a CTAB method and amplified using a nested PCR approach with group-specific primers AU2-AU4 and FF390-FR1GC [31,32]. The amplified PCR products were separated in 8% (*w*/*v*) polyacrylamide gel (acrylamide/bis solution, 37.5:1) with a linear gradient of 25–55% denaturant using a Cipher DGGE Electrophoresis system (CBS Scientific, Del Mar, CA, USA) [33]. Phoretix 1D Pro Gel Analysis (Totallab, Newcastle upon Tyne, UK) and Primer version 7 (Primer-E Ltd., Plymouth Marine Laboratory, Plymouth, UK) were used to analyze the endophytic fungal communities as previously described [34]. Each band in the DGGE gel was considered as one fungal taxon. Fungal diversity was assessed based on the presence or absence of the same bands in different samples in DGGE gels. At the same time, fungal richness was assessed based on the number of bands per lane in the DGGE gel.

### 2.3. Isolation and Identification of Cultured Endophytic Fungi

Emerging fungal hyphae from *P. colorata* tissue sections plated on SNA plates were sub-cultured onto PDA plates by hyphal tipping using a sterile needle. The DNA for each isolate was extracted using the PureGene kit (Qiagen, Hilden, Germany) as per the manufacturer’s instructions, and the ITS gene was amplified using the primer pair ITS1 (5′-TCC GTA GGT GAA CCT GCG G-3′) and ITS4 (5′-TCC TCC GCT TAT TGA TAT GC-3′) [35]. The PCR-amplified ITS regions were sequenced, and the sequences were trimmed using DNAMAN v4 (Lynnon Biosoft, San Ramon, CA, USA). The final sequences were compared against known sequences in NCBI BLAST and were deposited in the GenBank database. Sequence alignment was performed using MUSCLE, distance matrices, and phylogenetic trees were calculated by neighbor-joining algorithms with 1000 bootstrap replication in Geneious prime software (Biomatters, Auckland, New Zealand).

### 2.4. Functional Activity of Endophytic Fungi Isolated from P. colorata

#### 2.4.1. Activity against Phytopathogenic Fungi

For the functionality testing, the endophytic fungi (*n* = 50) isolated from *P. colorata* were screened against phytopathogenic fungi *Neofusicoccum luteum* isolate ICMP 16678, *Neofusicoccum parvum* isolate MM562, *Ilyonectria liriodendri* isolate WPa1c, and *Neonectria ditissima* isolate ICMP 14417. *Neofusicoccum luteum* ICMP 16678 and *Neonectria ditissima* ICMP 14417 were obtained from the International Collection of Microorganisms from Plants (ICMP, Maanaki Whenua-Landcare Research, Lincoln, New Zealand), and *N. parvum* MM562 and *Ilyonectria liriodendri* WPa1c were obtained from Lincoln University Plant Microbiology culture collection. Activity against phytopathogenic fungi was tested on a dual culture on Waksman agar (WA) plates [19]. The antagonistic potential of the endophytic fungi was classified based on the zone of inhibition where high activity (zone of inhibition > 10 mm), moderate activity (zone of inhibition < 10 mm but >/=5 mm) and low activity (zone of inhibition < 5 mm but >2 mm).

#### 2.4.2. Activity against Opportunistic Human Pathogens

To assess the potential of endophytic fungi to produce antimicrobial compounds, potentially including polygodial like the host *P. colorata*, the endophytic fungi (*n* = 50) were tested against the opportunistic human pathogens *Staphylococcus aureus*, *Escherichia coli*, and *Candida albicans* in dual culture assays. The test isolates were obtained from the Institute of Environmental Science and Research (ESR, Porirua, New Zealand). GelAir Cellophane (Biorad) membranes were cut with a scalpel using a petri dish lid as a reference. The membranes were autoclaved prior to usage, and each membrane was carefully transferred onto individual WA plates using sterile forceps. The membrane was gently pressed onto the agar using a sterile spreader to ensure that the membrane adhered to the agar. Using a sterile cork borer, a 6 mm plug from the margins of a 3–5 d old test fungal colony was transferred onto the center of the cellophane membrane on the WA plates. The plates were sealed and incubated for 7 d at 25 °C in a 12 h light/12 h dark cycle. After 7 d, the cellophane membranes with the fungal mycelium were carefully lifted using sterile forceps and discarded. Using a sterile spreader, 100 µL of the overnight cultures of *S. aureus*, *E. coli*, and *C. albicans* grown in nutrient broth (NB, Difco laboratories, Detroit, MI, USA) were spread onto their respective test plates. A 100 µL aliquot of *S. aureus*, *E. coli*, and *C. albicans* was plated onto individual Waksman agar plates without any fungi growth on it as controls. The plates were sealed with parafilm and incubated at 25 °C for 24–48 h. The presence of a clear zone of inhibition in the plates was noted as being positive for inhibitory activity, and the results were recorded compared to control plates.

### 2.5. Influence of Endophytic Fungi on the Growth of P. colorata Seedlings in the Glasshouse

To assess the influence of endophytic fungi on the growth of *P. colorata* seedlings, selected isolates were applied as root/soil drenches to six-week-old *P. colorata* seedlings (Southern Woods Plant Nursery, Christchurch, New Zealand). The seedlings lacked a fully developed root system at the time of purchase and were acclimatized in the shade house for 3–4 weeks in February 2017 before the experiment. For inoculation, the 5–7 d old culture was grown on PDA, and when the fungal mycelia covered more than half of the agar surface, 1–2 mL SDW (sterile distilled water) was added to the plates. Using a sterile glass spreader, the spores were dislodged and the spore suspension decanted into a 50 mL tube. The spore concentration of the suspension was determined using a hemocytometer and adjusted to 1 × 10^5^ spores/mL in a total volume of 1 L of SDW in sterile aluminum trays (270 mm W × 95 mm H × 325 mm L). Prior to inoculation with the test fungal cultures, the seedlings were not watered for 24–48 h, and the length of the shoots was measured using a digital caliper. For treatment with the fungal spore suspensions, the *P. colorata* seedlings with their soil-plugs intact were carefully soaked in the spore suspensions overnight in the aluminum trays, with the trays being covered with cling wrap overlaid with aluminum foil to avoid evaporation and cross-contamination. The following day, the seedlings with the soil-plugs were repotted into 1 L pots containing a potting mix medium of [20% pumice, 80% composted bark, 2 kg/m^3^ Osmocote^®^ standard 3–4 months gradual release fertilizer (NPK 16-3.5-10 plus trace elements)], 1 kg/m^3^ agricultural lime, and 500 g/m^3^ Hydraflo^®^ 2 (granular wetting agent, Scott Australia Pty Ltd., Auckland, New Zealand). Each treatment was replicated 10 times, and 10 uninoculated control *P. colorata* seedlings soaked in SDW were also set up. The trial design in a randomized complete block design and the plants were watered once daily and regularly observed to see if there were any dead or diseased plants following the treatments. The plants were grown under a 12 h daylight and 12 h dark regime using an HPS lighting system (high pressure sodium) at 100 lumens light intensity.

After 3 months of growth (March 2017 to May 2017), the potting mix around the root region of each treatment of *P. colorata* seedlings was reinoculated by drenching with 50 mL of freshly prepared spore suspensions (1 × 10^5^ spores/mL) of their respective treatments. Four weeks after the second inoculation (June 2017), the seedlings were destructively harvested. At harvest, the shoot height was measured from the stem base (at the soil level) to the top leaf using a ruler. The number of internodes was measured for each plant stopping at the top two leaves. The difference in heights pre-treatment (X) and post-treatment (Y) was calculated (Y − X). The shoot and root portions were weighed after drying in an oven at 60 °C for 2 d. The data were analyzed using a general analysis of variance (ANOVA). Fisher’s protected least significant difference (LSD) was used to test the mean difference between shoot lengths, shoot weights, root weights, and the number of internodes of treated plants with untreated controls. The analyses were performed in Minitab 17 (Minitab LLC, State College, PA, USA).

### 2.6. Confirmation of Endophytic Colonization Using DGGE

To confirm whether the endophytic fungi were able to colonize the roots of *P. colorata* seedlings, after harvest a small section of the roots from treated and untreated control *P. colorata* seedlings were surface-sterilized as previously described and plated on PDA amended with ampicillin (100 µg/mL) to re-isolate the inoculated fungi. In addition, small sections of the roots were set aside for DGGE. PCRs and DGGE were performed as described previously. Pure DNA of endophytic fungal isolates used for inoculation experiments were also amplified using the same primer pairs and were used as reference markers in DGGE gels. The presence of the corresponding band in treatments indicated successful colonization by the endophytic fungi.

## 3. Results

### 3.1. Analysis of the Structure and Richness of Endophytic Fungi Using DGGE

**Figure 2:**
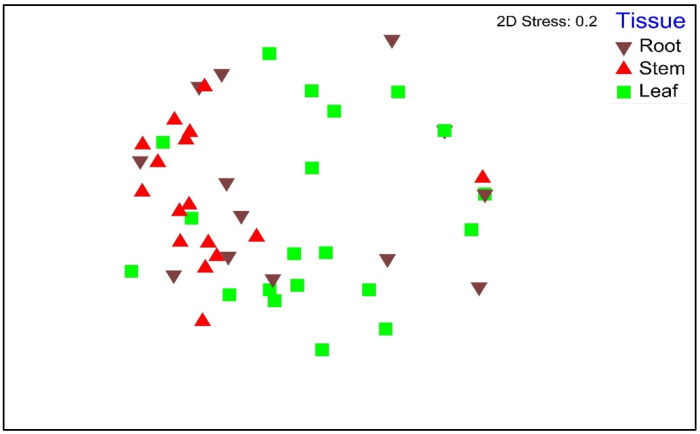
Nonmetric multidimensional scaling (MDS) plot showing endophytic fungal communities from different plant organs of *Pseudowintera colorata*.

Plant organ, location, and the interaction between the two factors influenced the endophytic fungal communities in *P. colorata* (PERMANOVA, *p* ≤ 0.005) (Table 2). The DGGE patterns grouped the endophytic fungal communities in the stems and roots together, whereas the fungal communities in the leaves were more diverse (Figure 2). The fungal taxa (*n*) were richer in the stems (*n* = 18) and roots (*n* = 15) compared to leaves (*n* = 5) (LSD, *p* ≤ 0.005). Plant location did not influence the richness of the fungal communities in *P. colorata* (Table 2).

### 3.2. Influence of Plant Age on the Community Structure and Richness of Endophytic Fungi in P. colorata

As the sampling sites chosen for this study were restricted to sites conserved by the New Zealand Department of Conservation (DOC), collecting plants of different ages in the same vicinity (+/−10 m) was not always possible. Thus, for analyzing the influence of plant age on the community structure and richness of endophytic fungi in *P. colorata*, a subset of three sites (Kaituna Valley Forest Park, Paringa Forest, and Peel Forest) where plants of different maturities were present was selected. For this study, plant height was used as a classifier, and the plants were classified as fully mature/old plants (>3 m) and young plants (≤1 m).

**Figure 3:**
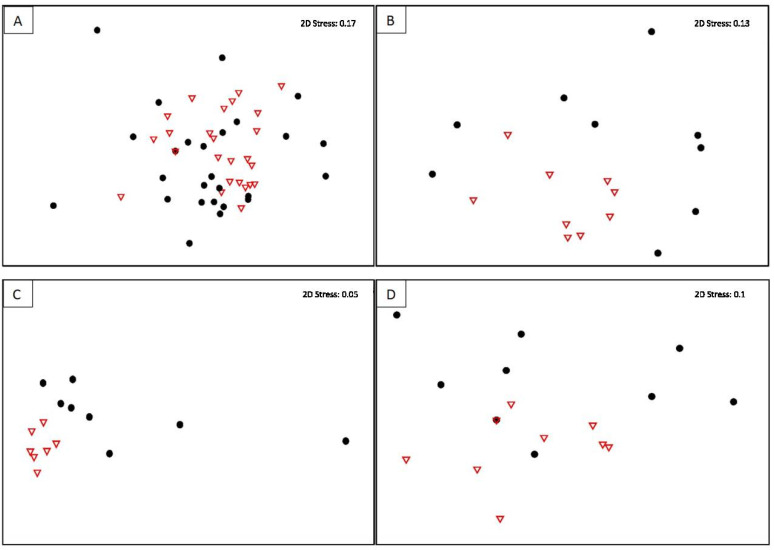
Nonmetric multidimensional scaling (MDS) plot showing endophytic fungal communities from old and young plants. (**A**) All plant organs, (**B**) roots, (**C**) stems and, (**D**) leaves of *Pseudowintera colorata*. Fully mature/old plant: ●; Young plant ∇.

All the factors and their interactions influenced the fungal communities in *P. colorata* (PERMANOVA, *p* ≤ 0.005) except for the interaction between location and plant maturity (Table 3). For the combined tissue data, the endophytic fungal communities were not distinguishable between mature and immature plants (Figure 3A). For individual organs, the endophytic fungal communities within the roots, stems, and leaves of the young plants formed discrete clusters (PERMANOVA; *p* = 0.002, *p* = 0.001 and *p* = 0.039, respectively), while the tissues of older plants were more diverse (Figure 3B–D).

### 3.3. Isolation, Sequencing Data, and Identity of Culturable Endophytic Fungi from P. colorata Tissues

A total of 200 endophytic fungi were isolated from the surface-sterilized *P. colorata* organs, which included 98 (49%), 80 (40%), and 22 (11%) isolates from leaves, stems, and root sections, respectively. The fungi (*n* = 200) were grouped into a representative set of 50 morphotypes (26, 18, and 6 from stem, roots, and leaves, respectively) based on their cultural characteristics on PDA, and were identified by sequencing the ITS gene (550–750 bp). The representative set (*n* = 50) was also used for bioactivity testing in dual culture assays. Following ITS gene sequencing analysis, 47 isolates were categorized to the genus level, whereas two isolates were identified to family level and one to order due to low sequence homology (Table 4). The sequences were deposited in NCBI with the accession numbers from OK036294 to OK036333 and MH844075 to MH844084.

### 3.4. Activity against Phytopathogenic Fungi and Opportunistic Human Pathogens

Of the total endophytic fungi tested (*n* = 50), ten isolates showed activity against at least 1 test pathogen (Table 5). *Pezicula* sp. PRY2BA2 and *Metarhizium* sp. PR1SB1 showed the highest activity and inhibited all the phytopathogenic fungi tested (Table 5). *Trichoderma* sp. PRY2BA21, and *Xylariaceae* sp. P4BB2 completely inhibited *C. albicans* in dual culture assays. *Pezicula* sp. PRY2BA2, *Fusarium* sp. P4LC2, *Phoma* sp. P1LA4, and *Chaetomium* sp. PR1BC2 produced clearance zones greater than 10 mm.

### 3.5. Influence of Endophytic Fungi on the Growth of P. colorata Seedlings

The inoculation of *P. colorata* seedlings with endophytic fungi significantly increased the growth for all the treatments compared to the control (*p* < 0.05) except for *Metarhizium* sp. PR1SB1 (Table 6). Mean shoot height of seedlings treated with *Trichoderma* sp. PRY2BA21 was 2.2 × longer (8.36 cm) than the control (3.72 cm) but was not significantly different from other treatments (Table 6). Shoot and root dry weights of the treated seedlings were not significantly different from that of the control (*p* = 0.88 and *p* = 0.31, respectively) (Table 6). Treatment with *Fusarium* sp. P4LC2 produced significantly more internodes (mean = 7) compared with all other treatments (means = 6.0–4.1) (*p* < 0.005).

### 3.6. Colonization of P. colorata Roots by Endophytic Fungi as Shown by DGGE

Attempts to re-isolate inoculated fungi using traditional re-isolation techniques were unsuccessful as none of the recovered fungi showed any cultural similarities to the inoculated isolates. Therefore, the endophytic colonization of the roots by the isolates was confirmed using DGGE and DNA from the inoculated isolates used as reference markers. Bands corresponding to *Metarhizium* PR1SB1, *Xylariaceae* sp. P4LA3 and *Fusarium* sp. P4LC2 were identified in the respective treatments (Figure S1). *Xylariaceae* sp. P4LA3 showed a complex profile with 2 bands (Figure S1). Bands for other fungal isolates were not observed in the treatments. There was no attempt to re-isolate the endophytes onto agar from the tissues of the *P. colorata* seedlings.

## 4. Discussion

This study analyzed the diversity and functional potential of endophytic fungi in a primitive and important native New Zealand medicinal plant. It was the first study to comprehensively examine the community structure and diversity of endophytic fungi in *P. colorata* using a combination of culture-dependent and culture-independent techniques. The molecular tool DGGE, targeting the ITS subunit as a taxonomic marker, was used to analyze the structure and diversity of the endophytic fungal communities in *P. colorata* tissues [36]. Plant organ type was the main factor influencing the composition and richness of endophytic fungi in *P. colorata* and indicated that it is an overriding factor in the formation of endophytic fungal communities in *P. colorata* tissues. Similar findings were reported in other studies where tissue type primarily influenced the diversity and community structure of endophytic fungi [34,37].

Interactions between organ type and plant age of *P. colorata* influenced the diversity of endophytic fungal communities. The endophytic fungal communities in the roots, stems, and leaves of young *P. colorata* plants grouped together, while those in older plants were more diverse, suggesting a shift in the fungal communities as the plants matured. These results are consistent with a study on *Pinus taeda,* where a difference in richness and diversity of the endophyte community between seedlings and adult plants was observed [38]. Chemotype variability in *P. colorata* was demonstrated, which could account for the differences in the endophytic fungal communities across various locations [39]. In this study, all the tissues of *P. colorata* sampled (root, stem, and leaves) hosted at least one culturable endophytic fungus. These results support the theory that all individual plants on earth are colonized by one or more endophytes [3]. In this study, the fungal taxa in the stems was higher than in the roots and leaves. Similar results were reported from the Indian medicinal plant *Madhuca indica*, where a greater diversity of fungi was observed in stems [40]. However, a similar study on the Chinese medicinal plant *Stellera chamaejasmae* reported a higher number of endophytic fungi in roots compared to above ground tissues (stems and leaves) [41]. This difference in fungal communities in *P. colorata* can be attributed to some extent to the presence of antifungal compounds in the leaves and stems, which may place high selection pressure on the fungal communities and requires further investigation.

The bioactive potential of a randomly selected representative (*n* = 50) endophytic fungi isolated from *P. colorata* was tested against phytopathogenic fungi as well as opportunistic human pathogens, including the yeast *C. albicans*, the target of polygodial. In this study, several endophytic fungi demonstrated very strong antagonistic activity against phytopathogenic fungi such as *Neofusicoccum luteum*, *N. parvum*, and *Neonectria ditissima,* and also against opportunistic human pathogens such as *S. aureus* and *C. albicans*. Sequencing the ITS2 subunit revealed that the bioactive fungi belong to the genera *Trichoderma*, *Pezicula*, *Fusarium*, *Metarhizium*, *Chaetomium,* and *Xylariaceae* sp. Several studies have demonstrated the potential of endophytic *Trichoderma* sp. and *Chaetomium* sp. as biocontrol agents against phytopathogenic fungi such as *Fusarium solani* and *Phytophthora nicotianae* [42,43].

Of the strains tested, 18% (*n* = 9) of isolates showed high activity (zone of inhibition > 10 mm) against *C. albicans*. Similar results were reported for the Brazilian medicinal plant *Bauhinia forficate*, where 34.3% of the endophytic fungi isolates showed activity against *S. aureus*, *E. coli*, and *Streptococcus pyogenes* [7]. In addition, endophytic fungal isolates from *B. forficata* showed activity against pathogens such as *Aspergillus*, *Cladosporium*, *Cryptococcus*, *Candida,* and *Salmonella* [44]. In this study, the fungi identified as belonging to the genus *Pezicula* exhibited strong activity against all the phytopathogenic fungi tested and the yeast *C. albicans* strain 3395. Previous studies have reported that certain species of *Pezicula* produce one or more lipopeptide antimycotics known as pneumocandins, and several other compounds such as (R)-Mellein Echinocandin A, which are recognized for their activity against *C. albicans*, *S. aureus,* and *Ustilago violacea* [45,46].

This study is the first to demonstrate that endophytic fungi isolated from *P. colorata* can positively influence the growth of *P. colorata* seedlings. Treating *P. colorata* seedlings with endophytic fungi resulted in increased plant heights and quantity of internodes for six and five treatments, respectively, compared to the control. These results are consistent with similar research where inoculation of the root zone of 4-week saplings of *Boswellia sacra* with the endophytic fungus *Preussia* sp. BSL 10 increased shoot length and internodes compared to the untreated control [47]. *P. colorata* seedling treated with *Trichoderma* sp. PRY2BA21 increased the height of seedlings but did not affect the root and shoot dry weight. Similar results were reported in *Miscanthus* x *giganteus,* where shoot lengths of plants treated with an endophytic *Trichoderma* sp. were longer than the controls but were not different in terms of root and shoot biomass [48]. The increased plant growth in similar research following inoculation with endophytic fungi has been attributed to the production of growth hormones such as auxins, which are produced by several fungi including *Trichoderma* sp., and transportation of nutrients by organic matter mineralization [49,50,51]. However, in this study the exact mechanisms responsible for the increase in plant growth were not studied.

Although endophytic colonization was not confirmed by re-isolation for any of the endophytic inoculants, pure cultures of the fungal inoculants were included as reference markers in DGGE gels along with the treatments and compared to uninoculated control to check for the presence or absence of the marker bands. Out of seven endophytic fungal inoculants used in this study, marker bands of three isolates viz. *Metarhizium* sp. PR1SB1, *Xylariaceae* sp. P4LA3, and *Fusarium* sp. P4LC2 were detected in their respective treatments. Whereas the marker bands of *Trichoderma* sp. PRY2BA21, *Trichoderma* sp. PRY3BC1, and *Chaetomium* sp. PR1BC2 were not detected in DGGE. As these isolates were isolated from mature *P. colorata* plants, this variation in the age may have also affected successful colonization and re-isolation in this case. Although some of the isolates did colonize the plants, they still affected the growth of *P. colorata* seedlings. These findings indicated that the effect of these treatments could be due to increased nutrient availability in the root zone, especially as these fungi are also commonly known to exist as free-living saprophytes or associated with the rhizosphere [52]. However, the precise mechanism of how these endophytic fungi improve the growth of *P. colorata* merits further investigation. The expected outcomes of such future studies will help develop efficient strategies for harnessing the full potential of endophytic fungi, and also advance understanding of the ecology of endemic native medicinal plants.

## 5. Conclusions

This research is the first study to show the diversity and functional potential of culturable endophytic fungi in *P. colorata* in a New Zealand medicinal plant. In addition to showing strong antagonistic activity against phytopathogens and opportunistic human pathogens, the application of selected endophytic fungi as soil-drench also showed a positive effect on the growth of *P. colorata* seedlings. These results indicate the strong potential for endophytic fungi to be used in agriculture as plant growth promoters, as potential biocontrol agents against phytopathogens, and as sources of novel bioactive compounds.

## Figures and Tables

**Table 1:** Sampling information.

Location	Coordinates
Taihape Scenic Reserve (North Island)	−39.67635° S 175.80560° E
Tongariro National Park (North Island)	−39.02237° S 175.71810° E
Kaimanawa Forest Park (North Island)	−38.94721° S 175.94370° E
Lake Rotopounamu Scenic Reserve (North Island)	−39.02656° S 175.73502° E
Kahurangi National Park (South Island)	−41.07224° S 172.59166° E
Paringa Forest (South Island)	−43.69379° S 169.40724° E
Arthur’s Pass National Park (South Island)	−42.94215° S 171.56414° E
Kaituna Valley Scenic Reserve (South Island)	−43.71655° S 172.7554° E
Peel Forest (South Island)	−43.91835° S 171.25934° E
Otago Peninsula Scenic Reserve (South Island)	−45.88184° S 170.58049° E

**Table 2:** Influence of location and plant organs on the similarity and richness of endophytic fungal communities in *Pseudowintera colorata*.

Treatment	Microbial Communities Similarity ^a^	Microbial Richness ^a^
Location	0.081	0.095
Plant organ	0.001 **	<0.001 **
Location vs. Plant organ	0.002 **	<0.001 **

^a^ Asterisk denotes levels of statistical significance of microbial community similarity based on PERMANOVA and microbial richness based on GLM. ** High significant difference (*p* ≤ 0.005).

**Table 3:** Influence of location, plant organ, and plant age on the similarity and richness of endophytic fungal communities in *Pseudowintera colorata*.

Treatment	Microbial Communities Similarity ^a^	Microbial Richness ^a^
Location	0.002 **	0.121
Plant organ	0.001 **	0.015 *
Plant age	0.002 **	0.785
Location vs. Plant organ	0.001 **	<0.001 **
Location vs. Plant age	0.164	0.448
Plant organ vs. Plant age	0.001 **	0.393
Plant organ vs. Location vs. Plant age	0.001 **	0.060

^a^ Asterisk denotes levels of statistical significance of microbial community similarity based on PERMANOVA and microbial richness based on GLM. * Significantly different (*p* ≤ 0.05), ** High significant difference (*p* ≤ 0.005).

**Table 4:** Identity of culturable endophytic fungi isolated from *Pseudowintera colorata* based on ITS sequencing.

Location	Plant Organ	NCBI Match	Identity (%)	Classification
Kahurangi Nat. Park	Stem	*Acrocalymma vagum* strain 29T (1) (KP784427)	100	*Acrocalymma* sp.
Kaituna valley scenic reserve	Root	*Alternaria tenuissima* isolate F293.82.1 (MZ678531)	100	*Alternaria* sp.
Kaituna valley scenic reserve	Root	*Chaetomium globosum* isolate 1 (FJ791145)	99	*Chaetomium* sp.
Kaituna valley scenic reserve	Stem	*Paraconiothyrium* sp. isolate MBD_4091 (MK595563)	98	*Paraconiothyrium* sp.
Kaituna valley scenic reserve	Stem	*Fungal* sp. isolate 81 (MT820053)	100	Ascomycota
Kaituna valley scenic reserve	Stem	*Aspergillius arcoverdensis* strain CBS DTO_316-F9 (KY808749)	100	*Aspergillus* sp.
Kaituna valley scenic reserve	Root	*Aspergillus parvulus* culture IBT:22045 (KX423655)	100	*Aspergillus* sp.
Arthur’s Pass nat. park	Root	*Myceliophthora verrucosa* (KM527251)	100	*Myceliophthora* sp.
Arthur’s Pass nat. park	Stem	*Chaetomium cupreum* (KM357332)	100	*Chaetomium* sp.
Tongariro nat. park	Root	*Chaetomium globosum* strain SYP-F8042 (MN960568)	100	*Chaetomium* sp.
Lake Rotopounamu	Root	*Chaetomium globosum* isolate UWR_157 (MN654349)	99	*Chaetomium* sp.
Kaituna valley scenic reserve	Root	*Cadophora* sp. BESC103j (KC007139)	100	*Cadophora* sp.
Kahurangi Nat. Park	Stem	*Cadophora* sp. BESC103j (KC007139)	100	*Cadophora* sp.
Kahurangi Nat. Park	Stem	*Cadophora* sp. isolate 500-G (MK163779)	100	*Cadophora* sp.
Kahurangi Nat. Park	Leaf	*Paraconiothyrium variabile* isolate BL (KR909137)	99	*Paraconiothyrium* sp.
Taihape scenic reseve	Root	*Paraphoma chrysanthemicola* strain HLP7 (MG025864)	100	*Paraphoma* sp.
Lake Rotopounamu	Stem	*Penicillium* sp. strain UNIJAG.PL.202 (MT357207)	100	*Penicillium* sp.
Lake Rotopounamu	Stem	*Penicillium brefeldianum* strain G26 (MT601953)	100	*Penicillium* sp.
Lake Rotopounamu	Stem	*Penicillium* sp. E/As/10/7 (JX238733)	100	*Penicillium* sp.
Kaimanawa forest park	Stem	*Periconia macrospinosa* strain ZMXR37 (MT446142)	100	*Periconia* sp.
Arthur’s Pass nat. park	Stem	*Pezicula ericae* isolate ARSL_190907.7	100	*Pezicula* sp.
Arthur’s Pass nat. park	Stem	*Pezicula ericae* (NR_155653)	99	*Pezicula* sp.
Taihape scenic reseve	Root	*Phoma* sp. NRRL 54108 (HM751088)	99	*Phoma* sp.
Tongariro nat. park	Root	*Phomopsis* sp. isolate 5(1)b (MT278345)	100	*Phomopsis* sp.
Kaimanawa forest park	Root	*Diaporthe columnaris* (MN540315)	99	*Phomopsis* sp.
Arthur’s Pass nat. park	Root	*Fusarium* sp. (MH550484)	100	*Fusarium* sp.
Taihape scenic reseve	Root	*Fusarium acuminatum* isolate N-51-1 (MT566456)	100	*Fusarium* sp.
Taihape scenic reseve	Root	*Fusarium tricinctum* strain ME4 (MK559443)	100	*Fusarium* sp.
Taihape scenic reseve	Stem	*Fusarium solani* isolate SY1 (MT605584)	100	*Fusarium* sp.
Tongariro nat. park	Stem	*Fusarium solani* isolate N-54-1 (MT560379)	100	*Fusarium* sp.
Taihape scenic reseve	Leaf	*Diplogelasinospora grovesii* CBS 340.73 (NR_077164)	94	*Diplogelasinospora* sp.
Taihape scenic reseve	Root	*Diplogelasinospora grovesii* CBS 340.73 (NR_077164)	94	*Diplogelasinospora* sp.
Peel forest	Leaf	*Diplogelasinospora grovesii* CBS 340.73 (NR_077164)	94	*Diplogelasinospora* sp.
Peel forest	Stem	*Clonostachys* sp. isolate RL478 (MT557564)	100	*Clonostachys* sp.
Peel forest	Stem	*Clonostachys rosea* isolate SRRB-171 (MT210883)	100	*Clonostachys* sp.
Peel forest	Stem	*Trichoderma* sp. (MT557215)	100	*Trichoderma* sp.
Peel forest	Stem	*Trichoderma koningiopsis* strain Tk1 (MT111912)	100	*Trichoderma* sp.
Lake Rotopounamu	Root	*Trichoderma viride* isolate CTs9 (MK290390)	100	*Trichoderma* sp.
Tongariro nat. park	Root	*Trichoderma spirale* isolate ELF14 (v)	100	*Trichoderma* sp.
Paringa forest	Stem	*Chaetomium* G7 (MG548563)	100	*Chaetomium* sp.
Peel forest	Leaf	*Fusarium tricinctum* (MH931273)	100	*Fusarium* sp.
Paringa forest	Stem	*Metarhizium* sp. (DQ385622)	100	*Metarhizium* sp.
Arthur’s Pass nat. park	Stem	*Pezicula neosporulosa* (LC206659)	100	*Pezicula* sp.
Paringa forest	Stem	*Pezicula* sp. 1 ICMP 18831 (JN225940)	100	*Pezicula* sp.
Peel forest	Leaf	*Phoma* sp. H39 (GU566295)	100	*Phoma* sp.
Arthur’s Pass nat. park	Root	*Hypocrea lixii* DAOM 229978 (EF191298)	100	*Trichoderma* sp.
Paringa forest	Stem	*Trichoderma* sp. XY24 (KX856006)	100	*Trichoderma* sp.
Paringa forest	Stem	*Trichoderma harzianum* strain I 33 (KT351798)	100	*Trichoderma* sp.
Peel forest	Stem	*Xylariaceae* sp. 5 ICMP 18786 (JN225905)	100	*Xylariaceae* sp.
Peel forest	Leaf	*Xylariaceae* sp. 5 ICMP 18786 (JN225905)	100	*Xylariaceae* sp.

**Table 5:** Antagonistic ctivity of endophytic fungi against phytopathogenic fungi (*Neofusicoccum luteum*, *N. parvum*, *Ilyonectria liriodendri*, and *Neonectria ditissima*) and human pathogens (*Candida albicans, Staphylococcus aureus*, and *Escherichia coli*) based on the inhibition zone size (+++ high activity, ++ moderate activity, + low activity, − no activity).

Isolate	*N. luteum*	*N. parvum*	*I. liriodendri*	*N. ditissima*	*C. albicans*	*S. aureus*	*E. coli*
*Chaetomium* sp. PR1BC2	++	++	++	++	+++	+++	−
*Fusarium* sp. P4LC2	++	++	+	+	+++	+++	−
*Metarhizium* sp. PR1SB1	+++	+++	+++	+++	-	+++	−
*Pezicula* sp. AF2	+++	+++	+++	+++	+++	+++	−
*Pezicula* sp. PRY2BA2	+++	+++	+++	+++	+++	+++	−
*Phoma* sp. P1LA4	+++	+++	+	+	+++	+++	−
*Trichoderma* sp. F3	+++	+++	++	++	++	+++	−
*Trichoderma* sp. PRY2BA21	+++	+++	++	+	+++	+++	−
*Trichoderma* sp. PRY2BC1	++	+++	++	++	+++	+++	−
*Xylariaceae* sp. P4BB2	++	++	+	+	+++	+++	−

**Table 6:** Response of *Pseudowintera colorata* seedlings to treatment with endophytic fungi after 4 months growth. Mean of 10 replicate plants per treatment.

Treatment	Shoot Height (cm)	Shoot Dry Weight (g)	Root Dry Weight (g)	No. of Internodes
*Chaetomium* sp. PR1BC2	7.35 ab	0.98	0.54	6.0 b
*Fusarium* sp. P4LC2	6.79 ab	1.10	0.59	7.0 a
*Metarhizium* sp. PR1SB1	4.99 cd	1.10	0.47	4.3 d
*Trichoderma* sp. PRY2BA21	8.36 a ^1^	1.14	0.68	6.0 b
*Trichoderma* sp. PRY3BC1	7.46 ab	0.95	0.72	4.8 cd
*Xylariaceae* sp. P4BB2	6.84 ab	0.99	0.62	5.3 bc
*Xylariaceae* sp. P4LA3	5.97 bc	0.93	0.69	5.8 b
Untreated Control	3.72 d	1.02	0.59	4.1 d
*p* Value	<0.001	0.88	0.31	<0.001
LSD	1.771	NSD	NSD	0.816

^1^ Means followed by the same letter are not significantly different based on least significant difference (LSD) at *p* = 0.05, NSD—not significantly different.

## Data Availability

Raw sequence data reported in this paper have been deposited in GenBank under the accession numbers OK036294 to OK036333 and MH844075 to MH844084.

## Supplementary Materials

The following are available online at https://www.mdpi.com/article/10.3390/microorganisms9122576/s1. Figure S1: DGGE band patterns of pure cultures of endophytic fungi strains used as markers and DGGE band patterns of fungal communities (lanes 12–27) obtained from the roots of *P. colorata* seedlings treated with the respective endophytes. Lane 12—blank; Lane 13—*Metarhizium* sp. PR1SB1; Lane14–16 treatment PR1SB1; Lane 17—*Xylariaceae* sp. P4LA3; Lane 18–20 treatment P4LA3; Lane 21—*Fusarium* sp. P4LC2; Lanes 22–24 treatment P4LC2; Lanes 25–27 control seedlings.
