# Supplementary material for: Fungal Communities in the Native New Zealand Medicinal Plant Pseudowintera colorata (Horopito) Are Determined by Plant Organ Type and Host Maturity with Key Members Promoting Plant Growth
Source: Microorganisms. 2021 Dec 13;9(12):2576. doi: 10.3390/microorganisms9122576 (PMC8709005; doi:10.3390/microorganisms9122576)
Supplement: Supplementary file 1 [file microorganisms-09-02576-s001.zip › microorganisms-1446196-supplementary.pdf]

microorganisms

MDPI

Article

Fungal Communities in the Native New Zealand Medicinal Plant *Pseudowintera colorata* (Horopito) Are Determined by Plant Organ Type and Host Maturity with Key Members Promoting Plant Growth

Neeraj Purushotham [1,*,†], Eirian Jones [1], Jana Monk [2] and Hayley Ridgway [1,3]

[1] Department of Pest-management and Conservation, Faculty of Agriculture and Life Sciences, Lincoln University, Lincoln 7647, New Zealand; eirian.jones@lincoln.ac.nz (E.J.); Hayley.ridgway@plantandfood.co.nz (H.R.)
[2] AsureQuality, Lincoln 7647, New Zealand; Jana.monk@outlook.com
[3] The New Zealand Institute for Plant and Food Research Limited, Christchurch 7608, New Zealand
* Correspondence: neeraj@loambio.com
† Current address: Loam Bio., Orange 2800, Australia

Supplementary Materials

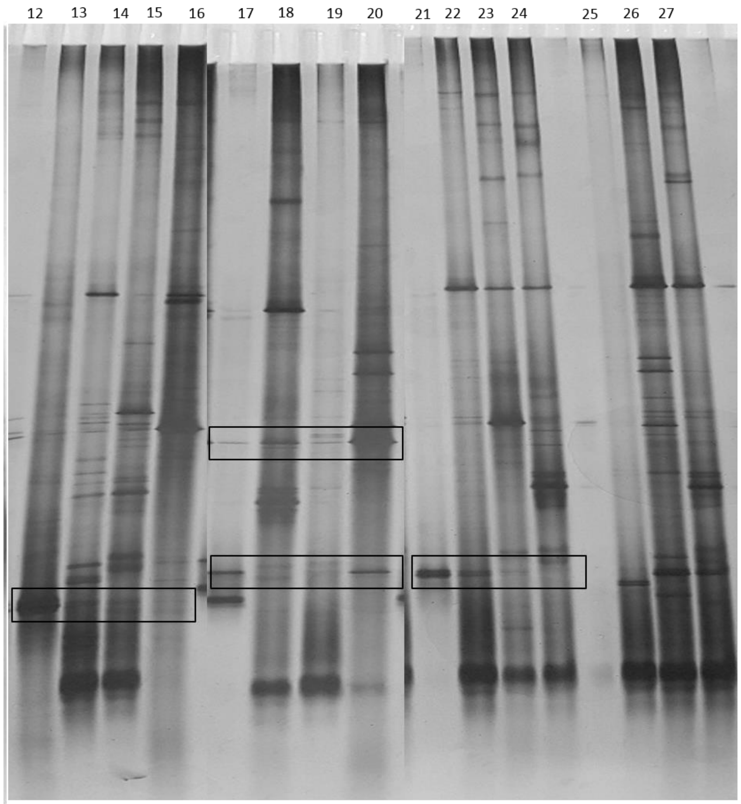

Figure S1. DGGE band patterns of pure cultures of endophytic fungi strains used as markers and DGGE band patterns of fungal communities (Lanes 12–27) obtained from roots of *P. colorata* seedling treated with the respective endophytes. Lane 12- blank Lane 13- *Metarhizium* sp. PR1SB1; Lanes 14–216 treatment PR1SB1; Lane 17- *Xylaria* sp. P4LA3; Lane 18–20 treatment P4LA3; Lane 21- *Fusarium* sp. P4LC2; Lanes 22–224 treatment P4LC2; Lanes 25–27 Control seedlings.
